# Distinct Cellular and Subcellular Distributions of G Protein-Coupled Receptor Kinase and Arrestin Isoforms in the Striatum

**DOI:** 10.1371/journal.pone.0048912

**Published:** 2012-11-06

**Authors:** Evgeny Bychkov, Lilia Zurkovsky, Mika B. Garret, Mohamed R. Ahmed, Eugenia V. Gurevich

**Affiliations:** Department of Pharmacology, Vanderbilt University Medical Center, Nashville, Tennessee, United States of America; INSERM/CNRS, France

## Abstract

G protein-coupled receptor kinases (GRKs) and arrestins mediate desensitization of G protein-coupled receptors (GPCR). Arrestins also mediate G protein-independent signaling via GPCRs. Since GRK and arrestins demonstrate no strict receptor specificity, their functions in the brain may depend on their cellular complement, expression level, and subcellular targeting. However, cellular expression and subcellular distribution of GRKs and arrestins in the brain is largely unknown. We show that GRK isoforms GRK2 and GRK5 are similarly expressed in direct and indirect pathway neurons in the rat striatum. Arrestin-2 and arrestin-3 are also expressed in neurons of both pathways. Cholinergic interneurons are enriched in GRK2, arrestin-3, and GRK5. Parvalbumin-positive interneurons express more of GRK2 and less of arrestin-2 than medium spiny neurons. The GRK5 subcellular distribution in the human striatal neurons is altered by its phosphorylation: unphosphorylated enzyme preferentially localizes to synaptic membranes, whereas phosphorylated GRK5 is found in plasma membrane and cytosolic fractions. Both GRK isoforms are abundant in the nucleus of human striatal neurons, whereas the proportion of both arrestins in the nucleus was equally low. However, overall higher expression of arrestin-2 yields high enough concentration in the nucleus to mediate nuclear functions. These data suggest cell type- and subcellular compartment-dependent differences in GRK/arrestin-mediated desensitization and signaling.

## Introduction

Upon persistent stimulation, many GPCRs undergo desensitization via a two-step process: activation-dependent receptor phosphorylation by G protein-coupled receptor kinases (GRKs) followed by the binding of arrestins that precludes further signaling via G proteins and induces receptor internalization [1], [2], [3]. Internalized receptor can either be recycled back to the plasma membrane or targeted for degradation, which leads to receptor down-regulation. Two of the four arrestins, arrestin-1 (a.k.a. rod) and arrestin-4 (a.k.a. cone), are expressed almost exclusively in the retina. The two non-visual arrestins, arrestin-2 (also known as ß-arrestin or ß-arrestin1) and arrestin-3 (ß-arrestin2 or hTHY-ARRX), are ubiquitous and apparently act on multiple GPCRs [4]. There are seven GRKs, four of which, GRK2 (historic name ß-adrenorenergic receptor kinase 1) and 3 (ß-adrenorenergic receptor kinase 2) (GRK2 subfamily) and GRK5 and 6 (GRK4 subfamily) are ubiquitously expressed in the brain [5], [6], [7], [8], [9]. Thus, four GRKs and two arrestins appear to be involved in the desensitization of hundreds of different GPCR subtypes.

Numerous data indicate that arrestin and GRK isoforms have important and highly specific functions in various physiological processes in the brain, as well as in neurological and psychiatric disorders (for review, see [2], [10]). However, despite their importance, many of even the most basic aspects of arrestin/GRK physiology in the brain remain unclear. Although the overall expression of arrestin/GRK isoforms has been documented for the rat [5], [8], [9], [11], monkey [12], and human brain [13], [14], the expression of arrestin and GRKs in different types of neurons has not been examined. Arrestins and GRKs are differentially targeted to subcellular compartments in neuronal cells [8], [13], [15], although the mechanisms responsible for very specific subcellular distribution of each isoform remain poorly understood. A better understanding of specific neuronal expression of each arrestin/GRK isoform will provide valuable insights into their functions. Differential cellular and subcellular distribution of arrestin/GRK isoforms is likely a critical determinant of their functional specificity considering that arrestins/GRKs demonstrate only limited molecular preference for particular GPCRs (for review see [2]). Thus, the sensitivity of GPCRs in different neuronal types and in subcellular compartments of neurons is likely differentially regulated depending on the cellular complement of arrestins and GRKs.

Here we report cellular and subcellular expression of arrestin-2 and -3, as well as GRK2 and GRK5 in the striatum. We compared the expression of arrestins and GRKs in direct and indirect pathway projection striatal neurons, as well as relative expression of arrestins and GRKs in striatal medium spiny neurons (MSN) and interneurons. We also examined the subcellular distribution of arrestins and GRKs in the human striatal tissue and determined the degree of nuclear localization of each isoform.

## Methods

### Ethics Statement

All animal procedures strictly followed guidelines in the Guide for the Care and Use of Laboratory Animals of the National Institutes of Health. The protocol was approved by the Institutional Animal Care and Use Committee of the Vanderbilt University (Permit Number: M-04-257). All surgery was performed under sodium pentobarbital anesthesia, and all efforts were made to minimize suffering. Human brain tissue was obtained from the Harvard Brain Tissue Resource Center (Director – Dr. F. Benes) supported by the National Institute of Health. The tissue collection was undertaken with the written approval of each subject and approved by the Institutional review Board of the Harvard University. The human samples were received coded by numbers without individual identifying characteristics.

### Animals and tissue preparation

Adult Sprague-Dawley rats (Charles River) were used for these experiments. The animals were housed in Vanderbilt University animal facility with 12/12 h light/dark cycle with free access to food and water. Rats were deeply anesthetized with pentobarbital (50 mg/kg i.p.) and transcardially perfused with saline followed by 4% paraformaldehyde. The brains were removed, postfixed overnight in the same solution, cryoprotected in 30% sucrose, frozen on dry ice, and kept at −80°C until needed.

### Human post mortem samples

The tissue from the caudate nucleus from control individuals without psychiatric or neurological disorders and no history of the use of psychotropic medication or drugs of abuse was available for analysis [16]. The age was 52.6±7.2 (mean±S.E.M.) years; post-mortem interval - 14.8±3.4 hours. Of 7 samples, 2 were from females and 5 from males.

### Tracer injection

Fluorogold (Molecular Probes, Carlsbad, CA) was injected into the substantia nigra pars reticulata at the coordinates AP = 5,4; L = 2,0; H = 8,3 from bregma; volume 0.3 µl at the rate 0.05 µl/min. Red latex RetroBeads (Lumafluor, Naples, FL) were injected into the globus pallidus at the coordinates AP = 0,9; L = 2,8; H = 6,6 from bregma; volume 0.2 µl at the rate 0.05 µl/min. The animals were allowed to survive for 2 weeks, then anesthetized with pentobarbital and transcardially perfused as described above. Sections (30 µm) were cut on a cryostat and collected in phosphate buffered saline (PBS).

### Subcellular fractionation

Subcellular fractions were prepared essentially as described [8], [13]. Briefly, approximately 25 mg of striatal tissue was homogenized in ice-cold 4 mM HEPES, pH 7.4, 0.32 mM sucrose, 1 mM EGTA buffer containing protease inhibitor cocktail and centrifuged at 1000×g for 10 min at 4°C to remove nuclei and large debris. Supernatant was centrifuged at 10,000×g for 15 min, and the pellet was lysed by hypo-osmotic shock in 9 volumes of ice cold 4 mM HEPES, pH 7.4, for 30 min. The lysate was centrifuged at 25,000 g for 20 min at 4°C to obtain synaptosomal membrane fraction (LP1) and crude synaptic vesicle fraction (LS1). Supernatant was centrifuged at 165,000×g for 2 hours to obtain cytosolic fraction (S3) and light membrane fraction (P3). The purity of the subcellular fractions was determined by the expression of specific markers.

The nuclear fraction was separated from non-nuclear contaminations using Nuclei Pure Prep Nuclei Isolation Kit for tissue (Sigma-Aldrich, St.Louis, MO) according to manufacturer's instructions. Briefly, the human caudate nucleus tissue was homogenized in 10 volumes of 4 mM HEPES, pH 7.4, containing 1 mM EGTA and 0.32 M sucrose, and the homogenate was centrifuged at 1,000 g for 10 min at 4°C. The supernatant (S1) consisted of non-nuclear subcellular compartments. The pellet (P1) containing nuclei and cell debris was resuspended in ice-cold Lysis solution (Nuclei PURE Lysis buffer containing 1 mM dithiothreitol and 0.1% Triton X-100). The sample was centrifuged through the 1.8 M sucrose cushion at 30,000 g for 45 min at 4°C. The nuclei-containing pellet was resuspended in Nuclei PURE storage buffer, washed once with the buffer, finally resuspended in 200 µl of the storage buffer and stored at −80°C until needed. The purity of the nuclear fraction was ascertained by microscopic analysis and by the use of fraction markers.

Protein concentration in all subcellular fractions was measured with Bradford reagent (Bio-Rad, Hercules, CA). Samples were then precipitated with 90% (v/v) methanol. The protein was pelleted by centrifugation [10,000×g, 10 min at room temperature (RT)], washed with 1 ml of 90% methanol, dried, and dissolved in sodium dodecyl sulfate sample buffer at the final concentration of 0.5 mg/ml.

### Immunohistochemistry and Western Blot

The sections were blocked for 1 h at RT in PBS containing 0.3% Triton X-100, 5% normal goat serum, and 3% bovine serum albumin. The rabbit polyclonal antibodies to GRK2 (sc-562; 1∶200) and GRK5 (sc-565; 1∶100) (Santa Cruz, CA) were used for immunohistochemistry. We have previously extensively characterized these antibodies for specificity [8], [11]. Arrestins were detected using rabbit polyclonal antibodies (1∶300) described previously [8], [9], [12], [13], [14]. To label different classes of interneurons, goat polyclonal antibody against choline acetyltransferase (ChAT) (Chemicon; 1∶20) and mouse monoclonal anti-parvalbumin (PV) antibodies (Sigma; 1∶500) were used. The sections were incubated with primary antibodies overnight at 4°C. After washing in PBS, sections were incubated with appropriate biotinylated secondary antibodies (1∶200, Vector Laboratories, Burlingame, CA) for 1 h at RT followed by incubation with streptavidin-Alexa 488 (1∶200, Molecular Probes) for 1 h at RT. For double labeling, arrestins and GRKs were visualized with biotinylated secondary antibody-streptavidin combination, and cell markers were visualized using appropriate secondary antibodies labeled with Alexa568 (red) and Alexa350 (blue). Fluorogold- and Alexa350-labeled cells were observed and photographed with DAPI filters, red Retrobeads and Alexa568 – with Cy3, and Alexa488 – with fluorescein filters. Nikon LP2000 microscope equipped with digital camera and QEG In Vivo imaging software (Media Cybernetics) was used.

For Western blots, samples were prepared as previously described [8], [12], [13], [14]. To evaluate the GRK5 subcellular distribution by Western blot, goat anti-GRK5 antibody from R&D Systems (Minneapolis, MN) (1∶500) and rabbit anti-GRK5 (sc-565; 1∶500) from Santa Cruz Biotechnology (Santa Cruz, CA) were used. Western blots were performed as described [8], [11]. GRK2 was detected with rabbit antibodies (1∶500) from Santa Cruz Biotechnology (Santa Cruz, CA). Arrestins were detected with arrestin-2- (1∶6,000) or arrestin-3-specific (1∶700) affinity-purified rabbit polyclonal antibodies as described [8], [9]. For quantification of arrestins, dilutions of standards containing 1∶1 mix of Escherichia coli-expressed purified bovine arrestin-2 and arrestin-3 in sample buffer were used. For quantification of GRKs, we used purified bovine GRK2 and human GRK5 as described [8], [12], [13], [14]. To verify the purity of subcellular fractions, the following antibodies were used: mouse anti-SNAP-25 (1∶1500, Millipore), mouse anti-PSD95 (1∶250, BD Biosciences), mouse anti-synaptophysin (1∶400, Sigma-Aldrich), mouse anti-Na^+^, K^+^-ATPase (1∶10,000, Millipore), rabbit anti-p53 (Cell Signaling Technology, 1∶1,000), mouse anti-histone H1 (Santa Cruz Biotechnology, 1∶500).

### Data Analysis

The images containing cells of interest were collected in the linear mode with the same camera settings using QEG in Vivo imaging software. For cell count and optical density measurements, IP Lab Imaging (BioVision Technologies) software was used. Measurements of the optical density of GRK staining in direct and indirect pathway neurons were made on the same sections. Multiple images were acquired from each section with 40× oil objective. Images selected for analysis contained several cells clearly labeled with Fluorogold and Retrobeads. Neurons that were not clearly labeled with either were ignored. Similarly, measurements of the optical density of GRK staining in medium spiny neurons in comparison to interneurons were made on the same triple-stained sections. Images selected for the analysis contained at least one ChAT-labeled, one or more PV-labeled neurons and multiple neurons lacking either ChAT or PV label (considered medium spiny neurons). Only neurons in the same plane of focus, through the nucleus with clearly visible outline of the nuclear membrane, were measured. Cell-free areas were measured on each image to provide the local background value that was subtracted from total optical density measurements for each image. Sections obtained from 3 rats were analyzed, 5–6 striatal sections per animal.

Statistical analysis of the data was performed using StatView software (SAS Institute, Cary, NC). The data for neuronal arrestin/GRK were analyzed by Student's t-test or one-way ANOVA with Cell Type as main factor followed by Bonferroni/Dunn post-hoc test with correction for multiple comparisons, where appropriate. In all cases, p<0.05 was considered significant.

## Results

### GRK and arrestin isoforms are equally abundant in direct and indirect pathways neurons in the rat striatum

**Figure 1:**
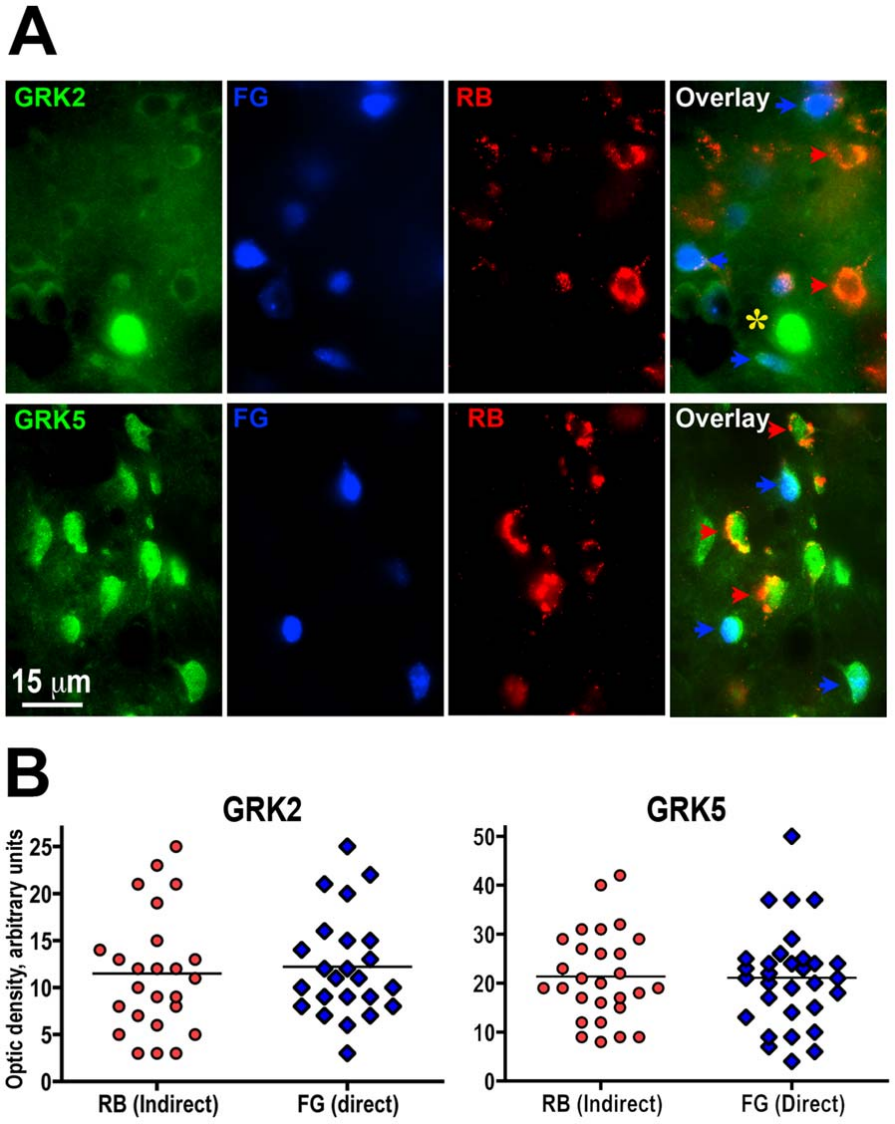
GRK isoforms are expressed in direct and indirect pathway striatal neurons at similar levels. (**A**) Photomicrographs show the expression of GRK2 (upper panels) and GRK5 (lower panels) in direct and indirect pathway medium spiny neurons. GRKs are labeled in green (left panels); direct pathway neurons were retrogradely labeled with fluorogold (FG, blue); indirect pathway - with Retrobeads (RB, red); right panels show an overlay of all three labels. Blue arrows points to examples of indirect pathways neurons expressing GRKs; red arrows point to examples of direct pathways neurons expressing GRKs; yellow star indicates a large presumably cholinergic interneuron. (**B**) Scatterplots of the optical density of GRK immunostaining in direct and indirect pathway neurons for GRK2 (left panel) and GRK5 (right panel). Horizontal lines correspond to the median values. There was no significant difference between the level of GRK2 or GRK5 in direct and indirect pathway striatal neurons.

We analyzed cellular distribution of GRK2 and GRK5 as representatives of the GRK2 and GRK4 subfamilies, respectively. We focused on the comparison of direct and indirect pathway striatal neurons. Figure 1A shows the expression of GRK2 and GRK5 in striatal neurons of the direct and indirect pathways, labeled with Fluorogold injected into the substantia nigra reticulata and with Retrobeads injected into the globus pallidus, respectively. Most cells in both direct and indirect pathways expressed GRK2 (98.5±1.1% and 96.3±0.8%, respectively). Similarly, GRK5 was expressed by 93.5±1.4% of cells of the indirect and by 99.2±0.5% of cells in the direct pathway. Thus, most striatal neurons of both types co-express GRK2 and GRK5. Quantification of the optical density on sections containing neurons labeled by retrograde tracers and processed for GRK immunohistochemistry yielded similar level of GRK2 signal in both direct and indirect pathway neurons (t(47) = 0.43, p = 0.67; Fig. 1B). Similarly, there was no significant difference between the GRK5 signal in direct and indirect pathway neurons (t(57) = 0.12, p = 0.91; Fig. 1B). These data show that the level of GRK2 and GRK5 expression is comparable in the direct and indirect pathway striatal medium spiny neurons.

**Figure 2:**
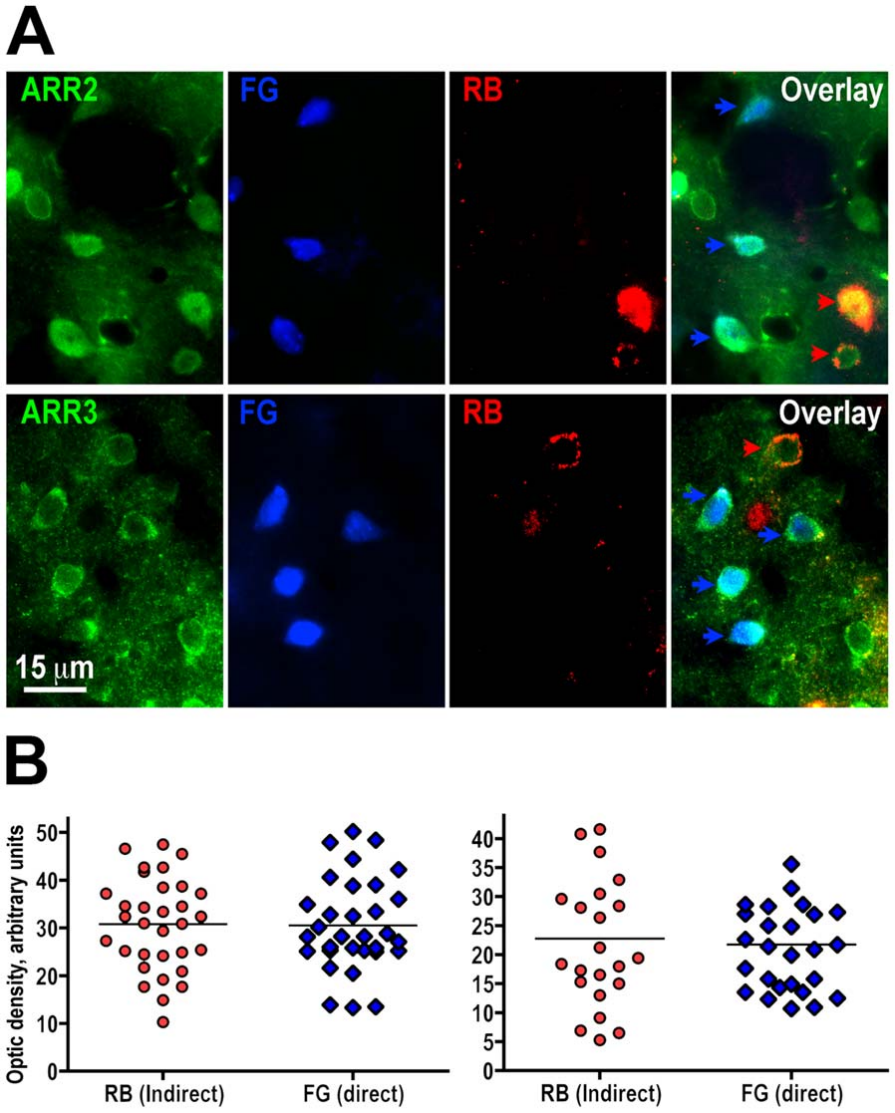
Both non-visual arrestins are expressed in direct and indirect pathway striatal neurons at similar levels. (**A**) Photomicrographs show the expression of arrestin-2 (ARR2) (upper panels) and arrestin-3 (ARR3) (lower panels) in direct and indirect pathway medium spiny neurons. Arrestins are labeled in green (left panels); direct pathway neurons - with fluorogold (FG, blue); indirect pathway - with Retrobeads (RB, red); right panels show an overlay of all three labels. Blue arrows points to examples of indirect pathways neurons expressing arrestins; red arrows point to examples of direct pathways neurons expressing arrestins. (**B**) Scatterplots of the optical density of arrestin immunostaining in direct and indirect pathway neurons for arrestin-2 (left panel) and arrestin-3 (right panel). Horizontal lines correspond to the median values. There was no significant difference between the level of arrestin-2 or arrestin-3 in direct and indirect pathway striatal neurons.

We have performed similar experiments aimed at establishing whether the expression of arrestin-2 and arrestin-3 differs between the direct and indirect pathway striatal neurons. Cell counts demonstrated that most cells in both pathways expressed arrestin-2 (98.5±1.1% and 96.3±0.8%, respectively). Similarly, arrestin-3 was expressed by 93.5±1.4% cells of the indirect and by 99.2±0.5% cells in the direct pathway (Fig. 2A). We found no significant difference in the level of expression of arrestin-2 between the direct and indirect pathway neurons (t(62) = 0.062, p = 0.93) (Fig. 2B). The level of arrestin-3 was also similar in direct and indirect pathway striatal neurons (t(48) = 0.37, p = 0.72) (Fig. 2B). Thus, MSN in both pathways express similar levels of the two main GRK isoforms and both non-visual arrestins.

### Differential expression of GRK and arrestin isoforms in interneurons

**Figure 3:**
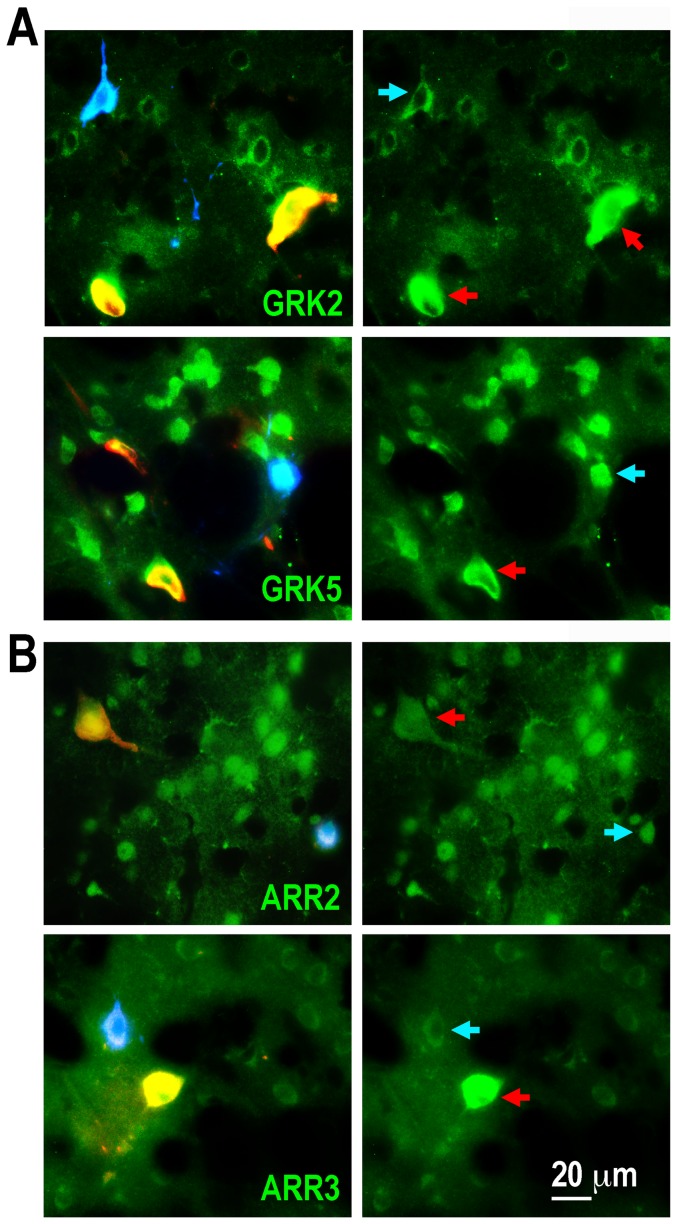
GRK and arrestin isoforms are differentially expressed in medium spiny neurons and interneurons in the striatum. Photomicrographs show the expression of GRK2 or GRK5 (**A**), and arrestin-2 (ARR2) or arrestin-3 (ARR3) (**B**) (green) in striatal neurons. GRK and arrestin isoforms were labeled with subtype-specific antibodies as described in Methods. Cholinergic interneurons were co-labeled with anti-choline acetyltransferase antibody (red; red arrows), and parvalbumin-expressing interneurons - with anti-parvalbumin antibody (blue; blue arrows). Cells negative for choline acetyltransferase and parvalbumin were considered medium spiny neurons. Note significantly higher expression of GRK2 in cholinergic and parvalbumin-expressing interneurons. Arrestin-3 is expressed at a higher level in cholinergic (but not parvalbumin-positive) interneurons.

**Figure 4:**
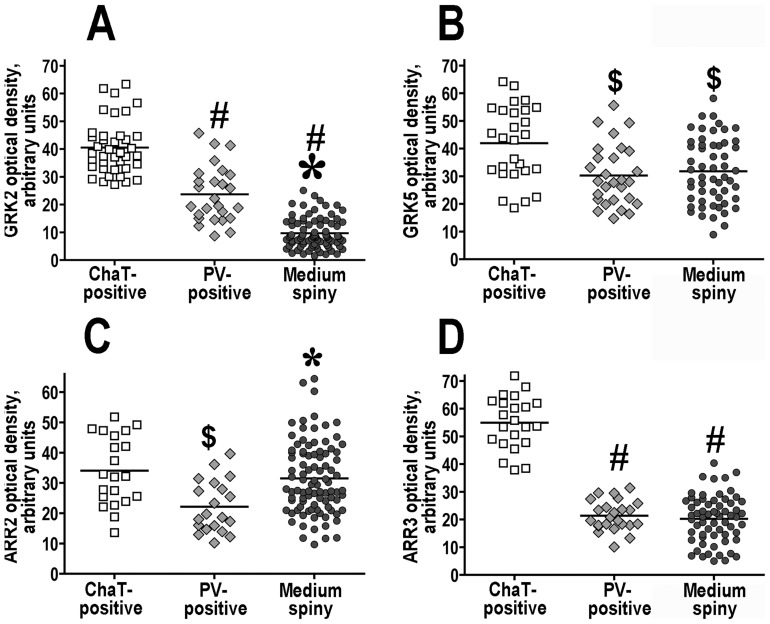
Cholinergic interneurons have the highest level of expression of GRKs and arrestins. Scatterplots of the optical density of GRK and arrestin immunostaining in medium spiny striatal neurons and interneurons for GRK2 (**A**), GRK5 (**B**) arrestin-2 (**C**), and arrestin-3 (**D**). Horizontal lines correspond to the median values. The quantification of the GRK or arrestin labeling density was performed on sections triple-stained for GRKs/arrestins, choline acetyltransferase (ChAT) (to label cholinergic interneurons), and parvalbumin (PV) (to label parvalbumin-positive interneurons). The data were analyzed by one-way ANOVA followed by Bonferroni-Dunn post hoc comparison. Large asterisk - p<0.001, small asterisk - p<0.01 to the PV group; pound sign (#) - p<0.001, dollar sign ($) - p<0.01 to the ChAT group according to the post hoc comparison.

Both arrestins and the two GRKs, 2 and 5, were also detected in striatal interneurons. GRK2 was particularly abundant in cholinergic interneurons (Fig. 3A, 4A), where its concentration substantially exceeded that in MSN. GRK2 was also significantly more abundant in another class of interneurons, PV-positive, although the GRK2 concentration in these cells was lower than that in cholinergic interneurons (Fig. 3A, 4A). In contrast, GRK5 was only slightly more abundant in cholinergic cells than in other neuronal types, and there was no difference in the GRK5 levels between MSN and PV-positive interneurons (Fig. 3A, 4B). The concentration of arrestin-2 was similar in cholinergic and MSN and significantly lower in PV-positive interneurons (Fig. 3B, 4C), whereas arrestin-3 was significantly more abundant in cholinergic neurons than in other neuronal types (Fig. 3B, 4D).

### Subcellular targeting of GRK5 is regulated by phosphorylation

**Figure 5:**
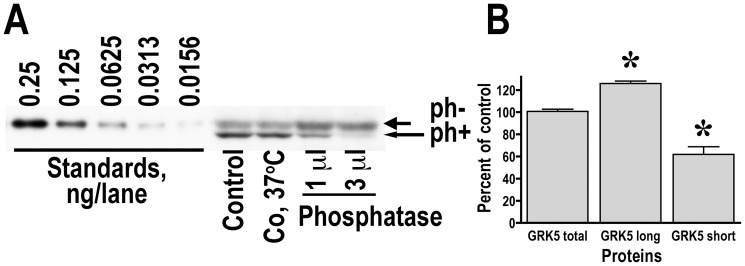
The lower band on the GRK5 blot represents a phosphorylated form of GRK5. (**A**) Representative Western blot showing the results of the phosphatase treatment of the human striatal samples. Samples were incubated with different amounts of alkaline phosphatase (calf intestinal, Promega) for 30 min at 37°C and blotted with the rabbit anti-GRK5 antibody sc-565 (Santa Cruz Biotechnology, Santa Cruz, CA) that shows high affinity to the lower band but detects both. Control – untreated sample, and Co, 37°C – a sample incubated for 30 min at 37°C without phosphatase. Note the disappearance of the lower band upon treatment and corresponding increase in the thickness of the upper band. (**B**) Quantification of four independent experiments (the values obtained with the lower amount of the phosphatase). * - p<0.05 as compared to Co, 37°C according to two-tailed Student's test.

In many brain areas, GRK5 was detected by Santa Cruz anti-GRK5 polyclonal antibody as a double band, whereas in other areas only the lower band was evident [8], [11], [14] (see also Fig. 5A). The more prominent lower band was positioned on the blot slightly lower than the standard purified GRK5. However, in the same samples anti-GRK5 goat antibody from R&D Systems consistently detected only one band that had the same apparent size as GRK5 standard [13]. GRK5 is subject to multiple posttranslational modifications. In particular, GRK5 autophosphorylates in a phospholipid- [17] and calmodulin-dependent [18] manner. GRK5 can also be phosphorylated by PKC [19] and by receptor tyrosine kinase PDGFRß [20]. Therefore, the lower band may represent a phosphorylated form of GRK5. To determine the identity of the bands, we treated human brain samples with alkaline phosphatase to dephosphorylate proteins. As shown in Figure 5A, 5B, progressive dephosphorylation resulted in disappearance of the lower band and an increase in the density of the slower running form. These results indicate that the lower band is indeed phosphorylated GRK5.

**Figure 6:**
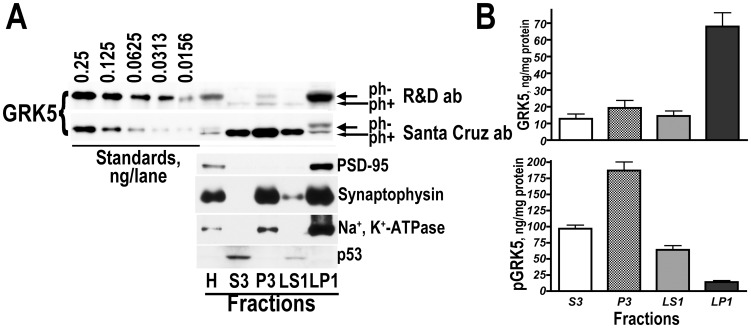
GRK5 phosphorylation alters subcellular distribution of the kinase. (**A**) Representative Western blot showing the detection of GRK5 in subcellular fractions from the human striatum with the anti-GRK5 antibody form R&D Systems (upper blot) or with the anti-GRK5 antibody from Santa Cruz Biotechnology (lower blot) (in the same samples). The R&D antibody has higher affinity for the upper band representing unphosphorylated GRK5, whereas Santa Cruz antibody has higher affinity for the lower band, i.e. phosphorylated GRK5. Note that unphosphorylated GRK5 localizes primarily to the synaptic membrane fraction (LP1), whereas phosphorylated GRK5 is abundant in P3, S3, and LS1 fractions. The standards contained indicated amounts of purified unphosphorylated GRK5; they provide a comparison of relative affinity of the antibodies to the unphosphorylated form of GRK5. (**B**) Quantification of the amount of GRKs 2 and 5 in the striatal subcellular fractions. Western blots were analyzed as described in methods. The values were converted into absolute units ng/mg proteins using purified GRK5 standards run on each blot. The values are for 7 independent samples of the human striatum.

Our data show that R&D antibody preferentially detects unphosphorylated GRK5, whereas Santa Cruz antibody detects phosphorylated GRK5 and unphosphorylated GRK5, albeit with lower affinity than R&D antibody. A comparison of the same GRK5 standards labeled with R&D (upper blot) and Santa Cruz (lower blot) antibody shown in Figure 5A demonstrates the difference in sensitivity between the two antibodies for unphosphorylated GRK5. To determine whether phosphorylation alters the subcellular targeting of GRK5, we analyzed the GRK5 content in subcellular fractions of the human striatum using both antibodies. As shown in Figure 6A, phosphotylated GRK5 (lower blot) is most abundant in the light membrane fraction (P3) as well as cytosolic (S3) and crude synaptic vesicle (LS1; which is also mostly cytosol) fractions. In contrast, unphosphorylated GRK5 is most abundant in the synaptic membrane fraction (LP1), with low presence in other subcellular fractions.

### Both GRK2 and GRK5 but not arrestins, are localized to the nucleus

**Figure 7:**
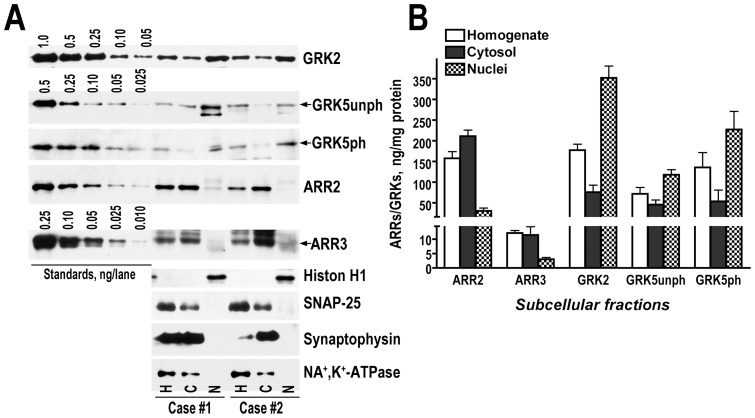
Both GRK isoforms are present the nuclei of striatal neurons. (**A**) Representative Western blot showing the detection of GRKs 2 and 5 in the nuclear (N) and cytosol, containing all non-nuclear elements including membranes, fractions (C) from the two cases of the human striatum. H - initial homogenate. The same amount of protein from all fractions was loaded on the gels (1 µg/lane for GRK2 and 2.5 µg/lane for GRK5). The standards contained indicated amounts of purified GRKs. (**B**) Quantification of the amount of GRKs 2 and 5 in the striatal subcellular fractions. Western blots were analyzed as described in methods. The values were converted into absolute units ng/mg proteins using purified GRKs standards run on each blot. The values are for 5 independent samples of the human striatum.

GRK5 has a nuclear localization sequence and was reported to bind DNA [21], whereas no such sequence has been found in GRK 2. To quantify precisely the amount of GRKs found in the nuclei, we isolated the nuclear fraction from the human caudate nucleus and determined the concentrations of GRKs in the nuclear and cytosolic fractions. Contrary to expectations, we found high concentrations of GRK2 and GRK5 in the nucleus as compared to the cytosol fraction (Fig. 7A, 7B). The level of GRK2 in the nucleus exceeded that in the cytosol by more than 4-fold (Fig. 7B). We detected both unphosphorylated and phosphorylated forms of GRK5 in the nucleus (Fig. 7A). The unphosphorylated GRK5 was enriched more than 2.5-fold in the nucleus as compared to the cytosol, and phosphorylated GRK5 - more than 4-fold, suggesting preferential nuclear localization of the phosphorylated form of GRK5.

In the same fractions we analyzed the distribution of arrestin isoforms between cytosol and nucleus. Arrestin-3, but not arrestin-2, has a nuclear export signal (NES) and has been shown to shuttle between the nucleus and cytosol [22], [23]. In contrast to GRKs, the level of arrestins in the nucleus was significantly lower than in the cytosol (Fig. 7A, 7B). Both arrestin isoforms showed similar distribution, with the level of arrestin-2 in the cytosol exceeding that in the nucleus by approximately 7-fold, and arrestin-3 - by 3.7-fold (Fig. 7B).

## Discussion

We surveyed the cellular and subcellular distribution of the two ubiquitous GRK isoforms, GRK2 and 5, and two arrestins, arrestin-2 and -3, in the striatum. One significant finding in this study is that most medium spiny striatal neurons co-express GRK2, GRK5 and both arrestins, and the level of expression of both GRKs and arrestins is comparable in direct and indirect pathway output striatal neurons. The direct and indirect pathway MSN express different complement of GPCRs. For example, dopamine D1 and D3 and A1 adenosine receptors preferentially localized to striatonigral, or direct, MSN, whereas D2 dopamine and 2A adenosine are segregated to striatopallidal, or indirect, MSN [24]–[29], see also [30] and references therein]. In the rat striatum, the D1 dopamine receptor preferentially interacts with arrestin-3 [31], whereas the D2 receptor - with arrestin-2 [32], although there is no difference in the arrestin levels between the D1- and D2-expressing MSN. Interestingly, in vitro D2 receptor binds both purified arrestin isoforms equally well [32].

We also found that cholinergic striatal interneurons express higher levels of GRK2, GRK5 and arrestin-3 than MSN. Another class of striatal interneurons, PV-positive GABAergic neurons, also differ in the expression of arrestins and GRK2 from the output neurons, being enriched in GRK2, but having lower levels of arrestin-2. Differential cellular expression of GRKs and arrestins may have important implications for the rate and extent of receptor desensitization and trafficking in different types of striatal neurons. Since there are only four ubiquitously expressed GRK isoforms and two non-visual arrestins, it is obvious that these proteins should have certain promiscuity towards GPCR subtypes. The degree of that promiscuity remains a matter of debate (for discussion see [2]). Experimental data *in vitro* and *in vivo* are somewhat inconsistent, with in vitro studies showing that arrestin/GRK isoforms are often highly interchangeable [6], [17], whereas studies with arrestin/GRK knockout mice demonstrate defined physiological function of each isoform [19], [33]–[39]. The level of expression of arrestins and GRKs has been shown both *in vitro* and *in vivo* to be an important determinant of the rate and extent of receptor desensitization and trafficking. Overexpression of arrestin and particularly GRKs in cultured cells invariably facilitates receptor desensitization and suppresses signaling, whereas reduction in the arrestin/GRK concentration impedes desensitization and enhances G protein-mediated signaling [40]–[44]. Similarly, *in vivo* lower concentration of arrestins and GRKs results in elevated signaling, whereas overexpression of these proteins has the opposite effect [33], [34], [36], [37], [38], [39], [45], [46]. Therefore, neurons with the overall lower availability of arrestins and GRK may have prolonged and/or enhanced signaling via specific GPCRs. Furthermore, depending on the cellular complement of arrestin and GRK isoforms, different receptors subtypes are likely to be differentially affected. For example, GRK2 function in striatal cholinergic interneurons has recently been shown to control behavioral responses to muscarinic receptor stimulation but not dopaminergic responsiveness [47] underscoring the cell-specific functional role of GRK isoforms. In addition to the action via phosphorylation-dependent mechanism, GRK2 is able to quench signaling of Gq-coupled GPCRs by binding and sequestering active GTP-liganded Gαq via their RGS homology (RH) domain [48]–[52]. GRKs other than GRK 2 and GRK3 do not seem to be able to interact with Gαq [48], [51], [53]. Thus, in neurons with abundant expression of GRK2, such as cholinergic striatal interneurons, the function of Gq-coupled GPCRs is likely to be controlled by GRK2 to some extent via a phosphorylation-independent RH-mediated mechanism, whereas in neurons with low level of GRK2 such mechanism is likely to play a less important role or none at all.

Another important finding in the study is the regulation of the GRK5 subcellular targeting by phosphorylation. We have previously reported using an antibody produced by Santa Cruz Biotechnology that GRK5 is most abundant in the light membrane fraction (P3) [8]. We later examined the subcellular distribution of GRK5 in the human striatal tissue with an antibody from R&D Systems that became available at the time, and found that GRK5 was most abundant in the LP1 [13]. It was unclear whether the discrepancy reflected the difference in the GRK5 subcellular distribution between the rat and human brain, which seemed unlikely, or if there was another reason. Here we resolved the controversy by demonstrating differential sensitivity of the antibodies we used to phosphorylated and unphosphorylated forms of the human GRK5 and establishing that unphosphorylated GRK5 is preferentially targeted to the synaptic membranes, whereas the phosphorylated form is retained in the light membrane fraction and even in the cytosol (Fig. 5). The GRK activity is regulated by phosphorylation by multiple kinases and autophosphorylation [17], [18], [54]–[59]. Phosphorylation of GRK2 by protein kinases A and C increases its kinase activity by facilitating the recruitment of GRK2 to the plasma membrane, possibly via GRK2 interaction with Gβγ that mediates GRK2 recruitment to active GPCRs [55], [59]. To the best of our knowledge, this is the first demonstration that phosphorylation of GRK5 affects its subcellular targeting. Furthermore, we show a shift in the GRK5 distribution upon phosphorylation between the synaptic and plasma membranes in striatal neurons in the brain. Generally, membrane anchoring of GRK5 is believed to be mediated by an amphipathic helix located in its C-terminal region [60]. However, such a mechanism cannot ensure specific localization to synaptic as opposed to any other membrane. Currently, there is no known mechanism that would mediate targeting to a specific membrane type. It is tempting to speculate that GRK5 interacts with proteins associated with the postsynaptic density, and this interaction is inhibited by GRK5 phosphorylation.

The current study also provides important data on the nuclear localization of GRK and arrestin isoforms. GRK5 is known to have a nuclear localization (NLS)/DNA binding signal [21]. Furthermore, GRK5 performs a physiologically important function in the nucleus by acting as a histone deacetylase kinase in cardiomyocytes [61]. Thus, it was reasonable to expect it to be abundant in the nucleus, and that is what we found. Surprisingly, GRK2 was also detected at high levels in the nuclear fraction (Fig. 7). In contrast to GRK5, GRK2 has no known NLS and no known function in the nucleus. However, the level of GRK2 in the nucleus, which is ∼3-fold higher in absolute values than that of unphosphorylated GRK5 and ∼1.6-fold higher than that of phosphorylated GRK5, suggests that there is a specific mechanism of GRK2 accumulation in the nucleus and that GRK2 may have yet undiscovered nuclear functions.

In contrast to GRKs, we found low levels of both arrestins in the nucleus as compared to other cellular compartments. These data are in agreement with our previous results demonstrating a low signal for arrestin-2 in the nuclei of rat striatal neurons detected by immunohistochemistry [15]. However, we also observed a higher arrestin-2 expression in other neuronal types, such as layer V pyramidal neurons in several cortical areas. Therefore, low nuclear levels of arrestin-2 may not be maintained in all neurons. Here we measured the level of arrestin-3 in the nucleus of striatal neurons for the first time and found it to be low. Arrestin-3 has an identifiable nuclear export signal (NES) [22], [23], shuttles between the nucleus and the cytoplasm, and relocalizes its binding partners such as Mdm2 and JNK3, from the nucleus to the cytosol in cultured cells [15], [62]. Arrestin-2 is also predominantly cytoplasmic in cultured cells. However, it does not contain an identifiable NES, and unless NES is engineered, it does not relocalize nuclear proteins to the cytosol [15], [62]. Recently, NLS has been identified in arrestin-2 [63]. Signaling functions of nuclear arrestin-2 have been reported [63], [64], [65], [66]. We found the proportion of arrestin-2 retained in the nucleus in the striatal neurons of the human brain to be slightly lower than that of arrestin-3 (14.5% versus 26%) (Fig. 7), which translates into ∼10-fold higher absolute level of arrestin-2 in the nucleus due to its overall higher expression. Therefore, although the bulk of arrestin-2 localizes to subcellular compartments other than the nucleus, the presence of arrestin-2 in the nucleus suggests a role for this isoform in nuclear functions as found previously in cultured cells [63], [64], [65], [66]. It is worth noting that so far no specific nuclear function of arrestin-2 has been identified in neuronal tissue in general and in the striatum in particular. Our data suggest that further experimentation is necessary to address this issue.

This first comprehensive study of cellular and subcellular distribution of the two main GRK isoforms and both non-visual arrestins yielded several novel observations. First, we found that the levels of GRKs and both arrestins in striatal MSN of both direct and indirect pathways are virtually the same. Second, we showed that two major types of striatal interneurons, cholinergic and PV-positive, express different complements of these key GPCR regulatory proteins. Third, we detected unexpectedly high levels of GRK2 in the nucleus, which exceed those of GRK5 with known NLS. Forth, we found that a higher proportion of arrestin-3 than arrestin-2 is localized to the nucleus, even though arrestin-3 has identified NES, whereas arrestin-2 does not. Finally, we detected fairly high absolute concentrations of arrestin-2 in the nuclei of striatal neurons, suggesting that it must have specific biological functions in this compartment.
